# Painless cheek nodule

**DOI:** 10.1016/j.jdcr.2024.01.005

**Published:** 2024-01-13

**Authors:** Yanci A. Algarin, Alice A. Roberts, Thomas W. Chu

**Affiliations:** a Department of Dermatology, Eastern Virginia Medical School, Norfolk, Virginia; b Department of Dermatology and Cutaneous Surgery, University of Miami Leonard M. Miller School of Medicine, Miami, Florida; c Department of Dermatology, Far Eastern Memorial Hospital, New Taipei, Taiwan

**Keywords:** face, Mohs micrographic surgery, primary cutaneous mucinous carcinoma, skin cancer, skin of color

## Case presentation

**Fig 1:**
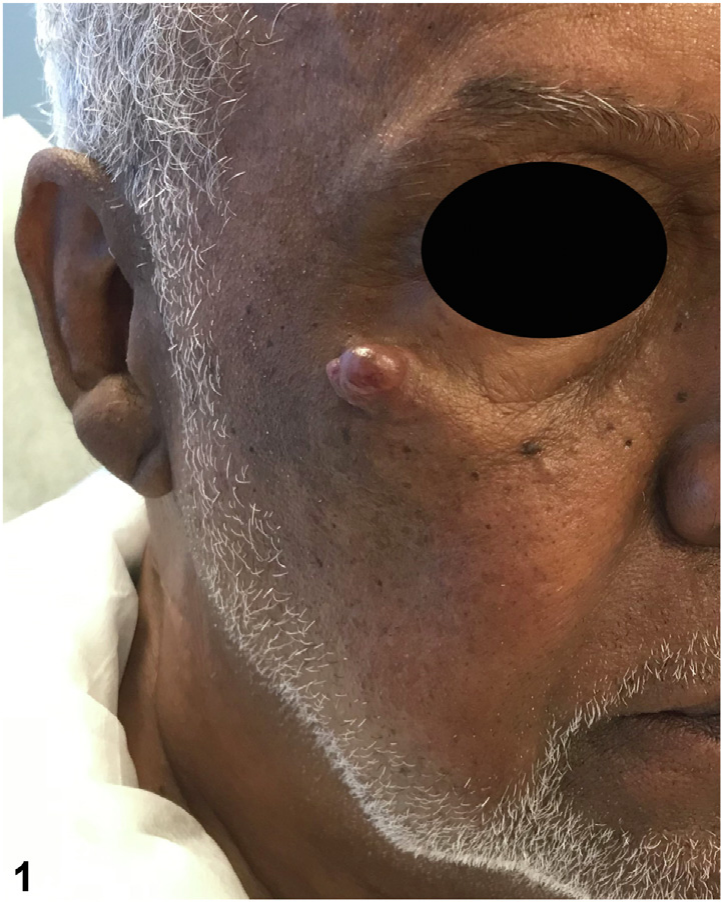

**Fig 2:**
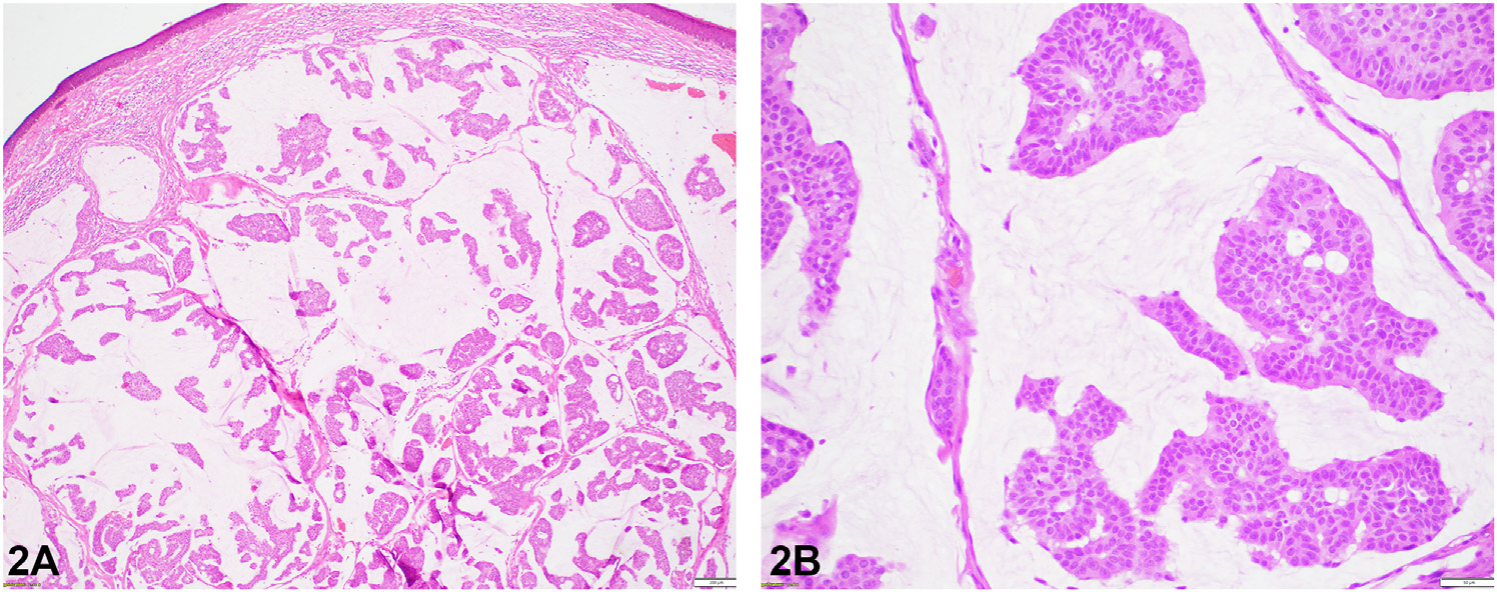

A 71-year-old man presented with a well demarcated, skin-colored nodule on the right zygomatic cheek present for 2 years that had slowly progressed in size. Physical examination revealed a 1.5 cm × 2.5 cm nodule with slight overlying erythema. The lesion interfered with the patient’s peripheral vision (Fig 1). He had no personal nor family history of skin cancer. He was otherwise in good health and review of systems was unremarkable. An incisional biopsy was performed with following immunohistochemical (IHC) staining (positive for CK7, P63, CK5/6, ER, and PR, and negative for CK20) and histopathologic analysis (Fig 2, *A* and *B*).

**Question 1: What is the most likely diagnosis?**

A. Basal Cell Carcinoma (BCC)
B. Chondroid Syringoma (CS) (mixed tumor)
C. Cutaneous Metastatic Adenocarcinoma
D. Primary Cutaneous Mucinous Carcinoma (PCMC)
E. Sebaceous carcinoma (SC)

**Answers:**

A. BCC – Incorrect. BCC is the most common cutaneous neoplasm, typically presenting as a slow-growing, painless “pearly” papules with telangiectatic vessels. Microscopic findings include basaloid cell islands with peripheral palisading, and a fibromucinous stroma. While mucin may be present, it usually surrounds tumor islands without forming large pools and does not characteristically form ducts.
B. CS – Incorrect. CS is a rare sweat gland tumor typically presenting as a slow-growing, painless, well-circumscribed, and variable colored (erythematous to bluish) nodule. Histologically, CS is characterized by a mix of epithelial and mesenchymal elements. The epithelial cells form ductal structures, while the mesenchymal component shows chondroid or myxoid stroma.
C. Cutaneous Metastatic Adenocarcinoma – Incorrect. Although it can mimic PCMC presenting as multiple, solid, painless nodules, the focal positivity for p63 and CK5/6 suggests PCMC. Negative CK20 staining essentially excludes a metastatic mucinous carcinoma of gastrointestinal origin,^1^ and imaging showed no primary malignancy.
D. PCMC – Correct. PCMC is a rare adnexal tumor of the sweat gland that most commonly affects the eyelid. Histopathological findings (islands of mildly pleomorphic cuboidal epithelial cells forming ducts and “floating” in mucin pools), IHC (positivity of p63, CK7, and CK5/6, combined with the negativity of CK20), and absence of metastasis support the diagnosis of PCMC.
E. SC – Incorrect. Although SC and PCMC may present with similar clinical features, they can be distinguished histologically. In SC, histological reveals dermal lobules typically composed of basaloid cells with areas of sebocytic differentiation, and commonly accompanied by observable mitotic figures and areas of necrosis.^2^

**Question 2: What should be considered for further evaluation or management in this case?**

A. Computed Tomography (CT) scan
B. No additional intervention is required
C. Colonoscopy
D. Magnetic Resonance Imaging (MRI) scan
E. Warm compresses

**Answers:**

A. CT scan – Correct. PCMC is often misdiagnosed clinically due to its unusual external presentation and microscopic similarity to cutaneous metastases originating from other mucinous carcinomas, such as those affecting the breast, gastrointestinal (GI) tract, lungs, ovaries, prostate, salivary glands, and lacrimal glands.^3^ Therefore, CT scans of the chest, abdomen, and pelvis are recommended to rule out metastasis.
B. No additional intervention is required – Incorrect. Morbidity related to PCMC is primarily associated with incomplete resection. While PCMC tends to grow slowly and have a good prognosis, cases of late recurrences and metastasis have been documented.^4^ Consequently, this is not an appropriate choice. Further assessment and definitive management, such as surgical excision or radiation therapy, are typically necessary to address the condition appropriately.
C. Colonoscopy – Incorrect. Colonoscopy is typically considered in the diagnostic workup of conditions like sebaceous carcinoma when there’s a suspicion of associated Muir-Torre syndrome. However, it is not a recommended step for the evaluation and management of PCMC. Instead, immunohistochemical staining can assist in differentiating tumor types.
D. Magnetic Resonance Imaging (MRI) – Incorrect. While MRI could estimate the size, location, and depth of the tumor, CT scans are recommended and necessary for assessing potential metastasis or involvement of internal organs.
E. Warm compresses – Incorrect. Warm compresses are inappropriate for PCMC management because they are typically used for conditions involving localized heat application to alleviate pain or inflammation, such as a hordeolum or chalazion in the eye.

**Question 3: What is the definitive treatment option in this case?**

A. Electrodessication and curettage (ED&C)
B. Mohs micrographic surgery (MMS)
C. Radiation therapy (RT)
D. Chemotherapy
E. Topical 5-Flurouracil (5-FU)

**Answers:**

A. ED&C – Incorrect. ED&C is a commonly used for warts or small superficial BCC and SCC in situ. However, it is generally not considered an appropriate treatment for PCMC due to its tendency to invade nearby tissue and metastasize, as well as its high recurrence rate. Additionally, ED&C has inadequate margin control.
B. MMS – Correct. Surgical removal is the definitive treatment option, with MMS preferred due to lower recurrence risk compared to standard excision. The use of MMS is associated with high cure rates, low recurrence rates, and optimal cosmetic outcomes.^4^
C. RT – Incorrect. PCMC is generally resistant to RT, and is not recommended for definitive treatment. However, postoperative adjuvant RT can be applied in recurrent cases of PCMC.^5^
D. Chemotherapy – Incorrect. Due to the localized nature of PCMC, chemotherapy, which is commonly used to target systemic cancers that have spread to different organs or lymph nodes, is not considered the standard treatment approach. Furthermore, recurrent PCMC is known to be resistant to chemotherapy.^3^
E. 5-FU – Incorrect. Surgical excision is the preferred treatment method for cutaneous malignancies. 5-FU is primarily used for treating actinic keratoses, superficial BCC, and SCC in situ, typically when patients are not suitable candidates for surgery.

## Conflicts of interest

None disclosed.
